# A Case of Cameron Lesions: An Overlooked Cause of Anemia in Patients With Gastrointestinal Bleeding and Hiatal Hernia

**DOI:** 10.7759/cureus.65510

**Published:** 2024-07-27

**Authors:** Pham Thao Vy Le, Hong Thoai Nguyen, Chau Dang, Khoa N Tran, Quynh Chau Vo

**Affiliations:** 1 Cardiovascular Research Laboratories, Methodist Hospital, Merrillville, USA; 2 Internal Medicine, Ascension Saint Joseph Hospital, Chicago, USA; 3 Internal Medicine, Desert Valley Hospital, Victorville, USA; 4 Cardiovascular Deparment, Yavapai Regional Medical Center, Prescott, USA

**Keywords:** blood transfusion, hiatal hernia, iron deficiency anemia, gastrointestinal bleeding, cameron lesion

## Abstract

Cameron lesions are rare causes of upper gastrointestinal bleeding (UGIB). The lesions are linear erosions or ulcers that develop in the sac of a hiatal hernia, which often go unnoticed in the upper gastrointestinal system, and are a prevalent cause of anemia resulting from iron deficiency. Postponed treatment can result in severe consequences such as potentially fatal hemorrhaging. Here, we present a case of a young woman who presented to the emergency room with recurrent gastrointestinal bleeding and severe microcytic anemia. The chest X-ray revealed a partial intrathoracic stomach, and a large hiatal hernia was subsequently confirmed in the CT scan of the abdomen and pelvis. The esophagogastroduodenal endoscopy indicated Los Angeles Classification System grade A reflux esophagitis and an 8 cm hiatal hernia with multiple Cameron ulcers with pigmented material and chronic non-erosive gastritis. Biopsies of the gastric body and antrum showed *Helicobacter pylori*-associated chronic active gastritis and intestinal metaplasia. An esophagus biopsy showed squamous esophageal mucosa with mild chronic inflammation. The patient was treated with a transfusion of three units of red blood cells, iron replenishment, and pantoprazole infusion and underwent hiatal hernia repair with mesh and Toupet fundoplication without any complications. After that, the patient was discharged and scheduled for follow-up with general surgery at the outpatient clinic.

## Introduction

Cameron lesions, first described in 1986 by Cameron and Higgins, refer to erosions or ulcers that form in the sac of a hiatal hernia and are frequently overlooked in the upper gastrointestinal system [1-3]. These lesions are believed to result from the compression of blood vessels by the diaphragm in a large hiatal hernia. Cameron lesions can induce prolonged bleeding, microscopic bleeding, or severe hemorrhage, leading to anemia due to iron deficiency, which accounts for 50% of all cases [4-6]. However, most participants had already undergone one or more upper endoscopies prior to their diagnosis. The management is dependent on the degree of clinical symptoms. Endoscopic hemostatic treatments are frequently employed for the management of acute hemorrhage [7]. In this report, we describe an uncommon case of a Cameron lesion in a young female patient with a large hiatal hernia. The patient experienced repeated episodes of bleeding and severe anemia but was effectively managed with blood transfusion, iron supplementation, and hernia repair.

## Case presentation

A 37-year-old woman with a history of gastritis and anemia, necessitating blood transfusions, presented to the emergency room with recurrent anemia and abdominal pain. The patient had no remarkable history of smoking, alcohol, recreational drugs, high stress levels, or dietary supplement intake. She had been experiencing burning epigastric pain for two years, exacerbated by eating and fasting, accompanied by nausea and non-bloody vomiting. Over the past two weeks, she reported symptoms of dizziness, lightheadedness, and dyspnea. Her primary care physician referred her to the emergency room due to severe anemia. On arrival, her vital signs were stable but she appeared pale, and her hemoglobin level was significantly low at 5.6 g/dl (12.0-15.3 g/dl). Additional laboratory results for the anemia workup are listed in Table 1. Her celiac disease panel was negative (Table 2). A peripheral blood smear confirmed microcytic anemia consistent with iron deficiency.

**Table 1:** Anemia workup MCV: mean corpuscular volume; MCH: mean corpuscular hemoglobin; MCHC: mean corpuscular hemoglobin concentration; UIBC: unsaturated iron binding capacity; TIBC: total iron binding capacity; LDH: lactate dehydrogenase

Component	Reference range	Result
Hemoglobin (g/dl)	12.0 - 15.3	5.6
MCV (fL)	80.0 - 100.0	56.7
MCH (pg)	26.0 - 34.0	15.9
MCHC (%)	32.5 - 35.8	28.0
Ferritin (ng/ml)	11 - 307	2
Iron (ug/dl)	50 - 212	12
UIBC (ug/dl)	155 - 300	> 450
TIBC (ug/dl)	228.0 - 428.0	Unable to calculate the value due to one or more analytes being outside the analyzer's reporting limit
Iron saturation (%)	20 - 55	Unable to calculate the value due to one or more analytes being outside the analyzer's reporting limit
Vitamin B12 (pg/ml)	180 - 914	286
Folate (ng/ml)	> 5.8	16.2
LDH	140 - 271	163
Total bilirubin (mg/dl)	0.0 - 1.0	0.3

**Table 2:** Celiac disease panel TTG: tissue transglutaminase

Component	Reference range	Result
IgA (mg/dl)	50-400	264
IgA endomysial antibodies screen	Negative	Negative
TTG IgA (Units)	< 20.0	< 1.9

**Figure 1:**
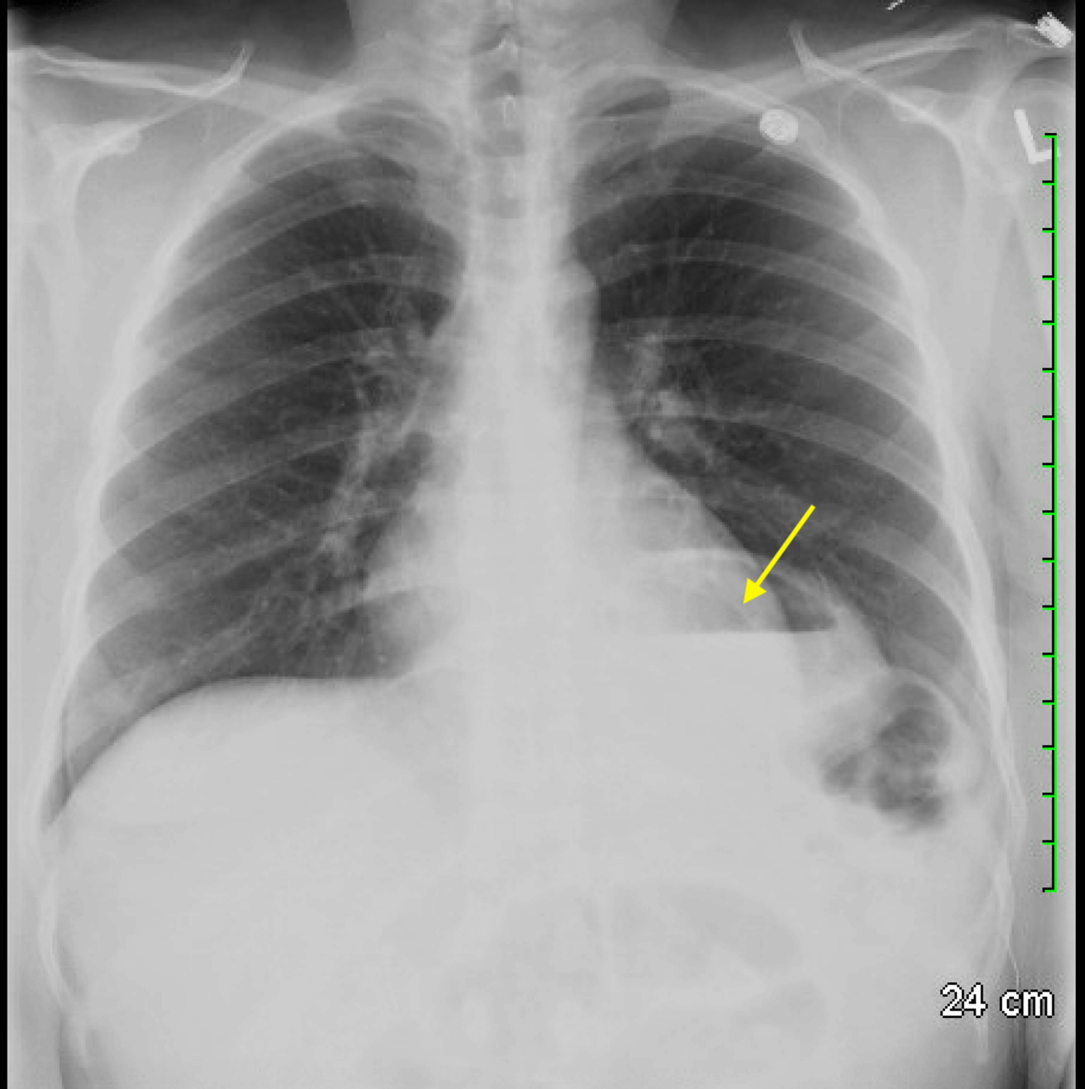
Chest X-ray showing a partial intrathoracic stomach (yellow arrow)

**Figure 2:**
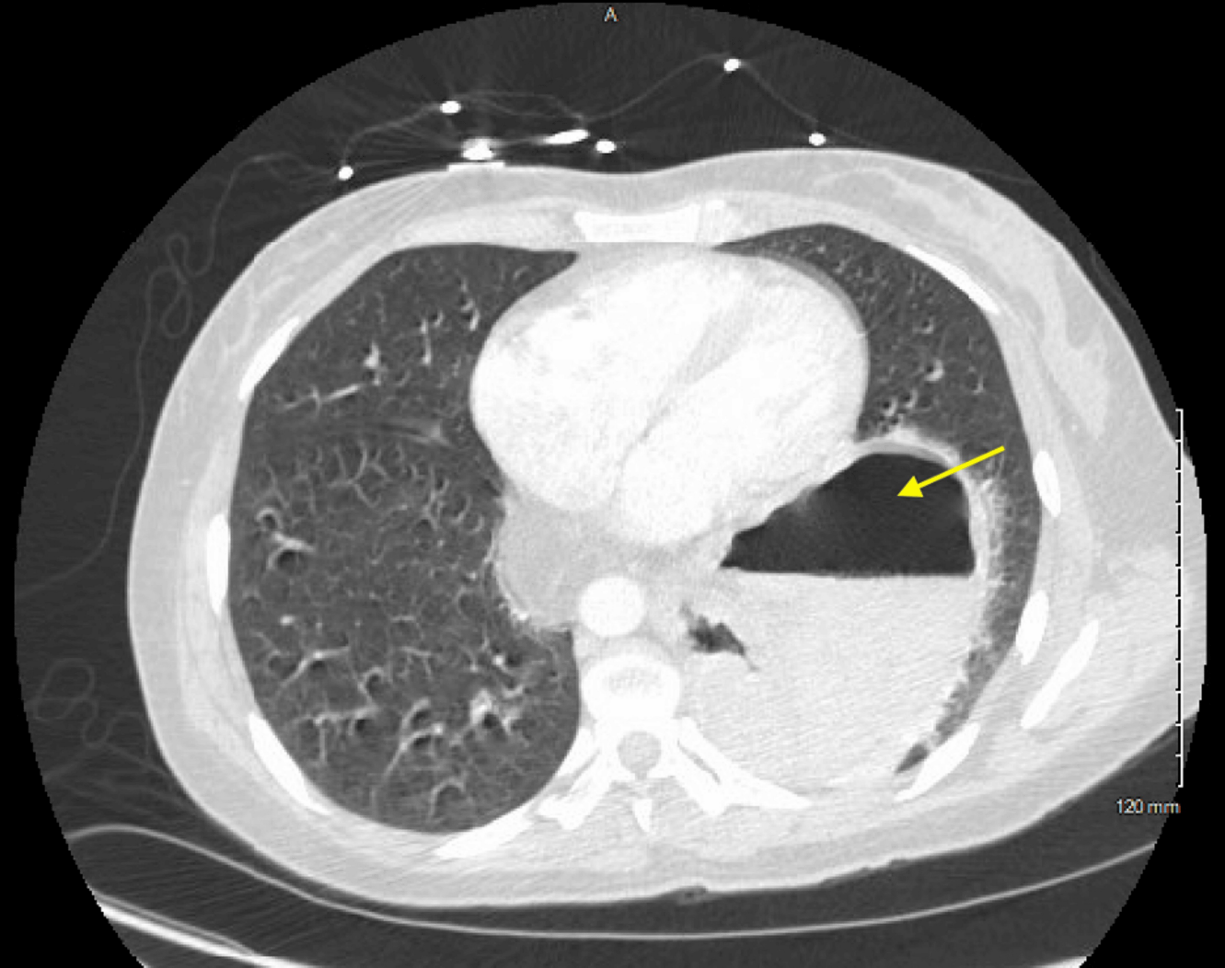
Axial cross-sectional CT scan of the abdomen and pelvis reveals a large diaphragmatic hernia at the hiatus. The stomach is quite dilated, with a prominent air-fluid level. Pylorus and the distal antrum are attenuated as they extend through the defect into the thorax (yellow arrow)

**Figure 3:**
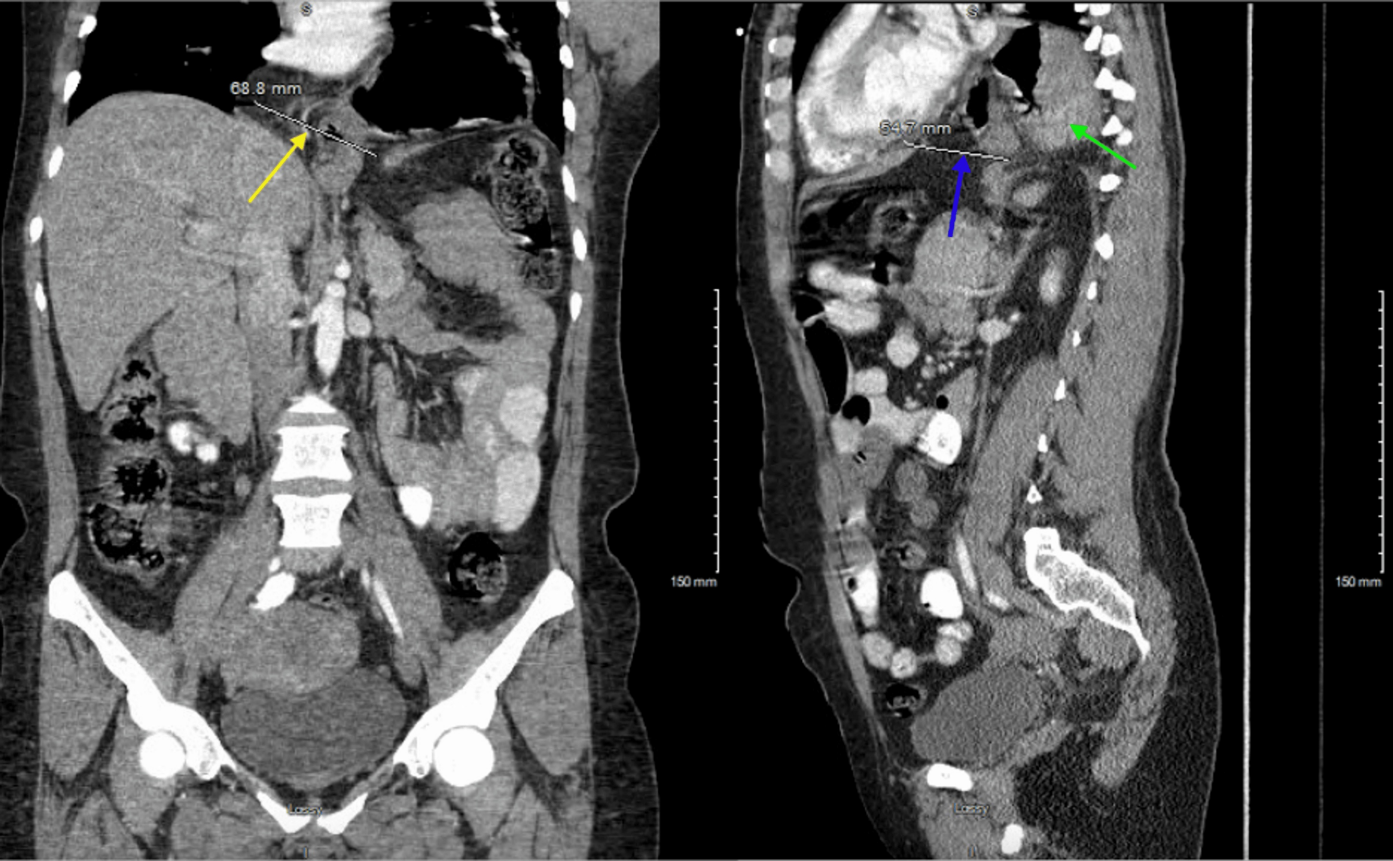
CT scan of the abdomen and pelvis shows a large diaphragmatic hernia at the hiatus. In a coronal section, the defect in the diaphragm is 7 cm in diameter, medial to lateral (yellow arrow), and in a sagıttal cross section, it is 5 cm in diameter, anterior to posterior (blue arrow). Most of the stomach is intrathoracic, with the antrum and distal stomach lying directly anterior to the fundus in a paraform (green arrow)

**Figure 4:**
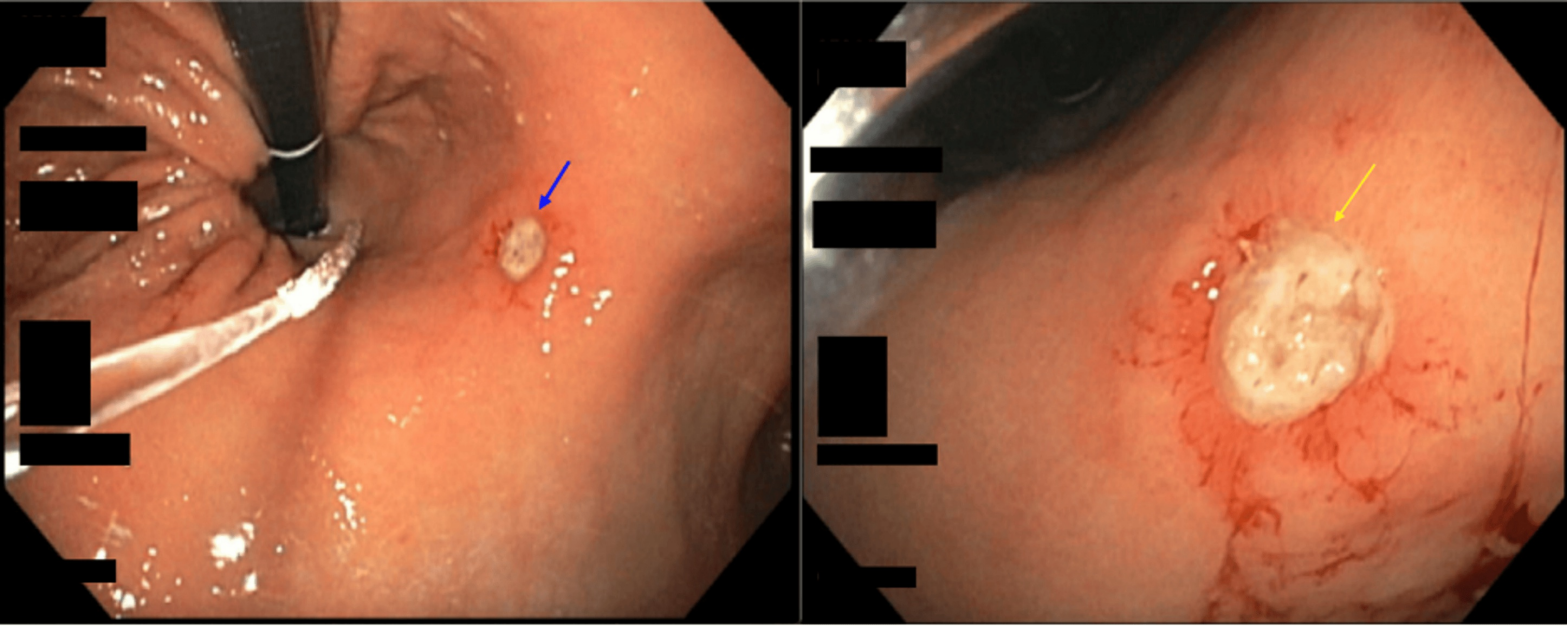
Esophagogastroduodenal endoscopy shows an 8-cm hiatal hernia grade IV according to the Hills classification with a Cameron ulcer (blue arrow) in the gastric body at the site of the hiatal hernia and one non-bleeding cratered gastric ulcer with pigmented material on the lesser curvature of the stomach (yellow arrow)

The patient's medical reports revealed a partial intrathoracic stomach in the chest X-ray (Figure 1) and a large para-esophageal hiatal hernia in the CT scan of the abdomen and pelvis (Figures 2, 3). The esophagogastroduodenal endoscopy indicated Los Angeles Classification System (LA) grade A reflux esophagitis with no bleeding, an 8 cm hiatal hernia grade IV according to the Hills classification with multiple Cameron ulcers, a non-bleeding gastric ulcer with pigmented material, and chronic non-erosive gastritis (Figure 4).

The colonoscopy did not detect any lesions. Biopsies of the gastric body and antrum showed H. pylori-associated chronic active gastritis and intestinal metaplasia. An esophagus biopsy showed squamous esophageal mucosa with mild chronic inflammation and no significant increase in eosinophils. Treatment included the transfusion of three units of red blood cells, a pantoprazole 40 mg infusion twice daily, and three doses of IV iron. The patient underwent robotic hiatal hernia repair with mesh and Toupet fundoplication without any complications. Additionally, the patient received treatment for *Helicobacter pylori* infection with doxycycline, metronidazole, and bismuth subsalicylate for 14 days. There was no postoperative drain placed in the patient. The patient was started on a clear liquid diet on day four after the operation and was not experiencing bloating or belching. On day six, the patient was discharged from the facility and scheduled for follow-up with general surgery at the outpatient clinic.

## Discussion

Cameron lesions are infrequent etiologies of upper gastrointestinal bleeding (UGIB). The term "Cameron lesions", initially introduced in 1986 by Cameron and Higgins, refers to erosions or ulcers that develop in the sac of a hiatal hernia and are commonly missed in the upper gastrointestinal tract [1-3]. Cameron lesions have been observed in approximately 5% of patients with a hiatal hernia who undergo upper endoscopy [3]. Additionally, they can result in persistent bleeding, occult bleeding, or overt hemorrhage, which can lead to anemia caused by a deficit of iron, which accounts for 50% of total cases [4-6].

While the exact cause is not fully understood, several variables that may contribute to the development of this condition include reflux, esophagitis, and mechanical trauma. These lesions are thought to arise from the compression of blood vessels by the diaphragm in a large hiatal hernia. The histopathologic alterations observed in the biopsy tissue of a Cameron lesion are a result of ischemia, which can be reversed by treating hiatal hernia [5-8]. This report presented a patient who experienced recurrent episodes of gastrointestinal bleeding and severe anemia that were subsequently managed with blood transfusions and iron supplementation. In order to directly examine the abnormality in the gastrointestinal tract, the next course of action would be to perform an endoscopy.

The diagnosis is established by directly observing the abnormality during the endoscopy. It is necessary to thoroughly examine the hiatal hernia as Cameron lesions can be linked to large type I hernias and be knowledgeable about the characteristic look of the abnormality, which is linear ulcers or erosions present on the mucosal folds of a hiatal hernia located at the diaphragmatic impression. Performing a biopsy is not advisable for diagnosing Cameron lesions [2]. In the present case, the CT scan of the abdomen and pelvis of the patient showed a large hiatal hernia near the esophagus, and the patient also had an esophagogastroduodenal endoscopy, which revealed an LA grade A reflux esophagitis and an 8 cm hiatal hernia with multiple Cameron ulcers, a non-bleeding gastric ulcer with pigmented material, and chronic non-erosive gastritis. 

Management, whether by conservative or surgical approaches, is contingent upon the severity of clinical manifestations and should be tailored to each patient on an individual basis. Due to the scarcity of information regarding their natural history and the limited number of patients, current medicine has been unable to establish standards for the treatment of Cameron lesions. Endoscopic hemostatic procedures are commonly used to treat acute bleeding [7]. Patients suffering from iron deficiency caused by persistent bleeding can receive treatment with a proton pump inhibitor following iron replenishment, which can potentially aid in preventing the recurrence of anemia [6]. Patients who continue to experience recurrent bleeding despite medical therapy may be candidates for surgery to repair the hiatal hernia, as seen in the patient in the current case, who received a blood transfusion, iron supplementation, and pantoprazole and underwent robotic hiatal hernia surgery with mesh and Toupet fundoplication. 

A literature review that searched 'Cameron lesions' and 'stomach' from January 2000 to July 15, 2020, yielded a total of 58 studies [6,8-15]. By analyzing these studies, it was seen that anemia was observed as a primary symptom in 62% of patients. Overt bleeding, characterized by hematemesis and/or melena, was present in 36% of patients. Hypovolemic shock was discovered in 2% of patients, whereas vomiting was observed in 1% of patients in these investigations. Furthermore, among the 45 patients whose data was presented, 31 of them (69%) had previously had one or more upper endoscopies before their diagnosis. Out of the total of 40 individuals, which accounts for 31% of the group, blood transfusions were necessary. Additionally, 12 people, equivalent to 9% of the group, required endoscopic hemostasis. Surgical intervention was needed by 37 individuals, making up 29% of the group.

The diagnosis of Cameron lesions is sometimes overlooked because they lack obvious signs and symptoms, have a hidden bleeding pattern, and are difficult to see during endoscopy, which poses a challenge for clinicians. When a patient has a hiatal hernia and experiences repeated gastrointestinal bleeding, it is important to examine the possibility of Cameron lesions, which should not be omitted if the initial endoscopy revealed no abnormalities. The endoscopic evaluation should include careful inspection of the hernia sac as well as the surrounding mucosa, using both forward and backward viewing to identify Cameron lesions, which are characterized as linear erosions inside the folds of the gastric mucosa. Delayed therapy may lead to serious effects, such as life-threatening bleeding. Another consequence includes the worsening of iron deficiency anemia and the deterioration of pre-existing comorbidities [16]. Additionally, it is necessary to distinguish between certain conditions that have similar clinical characteristics to Cameron lesions such as telangiectasias, ulcers, esophagitis, Mallory-Weiss tears, diverticulosis, Dieulafoy lesions, and neoplasms [17]. Utilizing an interprofessional healthcare team composed of doctors, nurse practitioners, physician assistants, nursing staff, and pharmacists working together as a collaborative unit is the most effective approach to attaining optimal outcomes in instances involving Cameron lesions.

## Conclusions

This report described the clinical presentation and effective treatment of Cameron lesions in a young female with a large hiatal hernia, recurrent gastrointestinal bleeding, and iron deficiency anemia, which is occasionally disregarded because of its non-specific symptoms. The study has contributed to existing literature and yielded new insights into the acute management of these patients. Postponed treatment can result in severe consequences, including potentially fatal hemorrhaging. The majority of individuals had already undergone one or more upper endoscopies before their diagnosis. Endoscopic hemostatic treatments are frequently employed for the treatment of acute bleeding. Patients with iron deficiency resulting from chronic bleeding can be treated with a proton pump inhibitor after replenishing their iron levels. The most effective method to achieve the best outcomes in cases involving Cameron lesions is to employ an interdisciplinary healthcare team that works together as a collaborative unit and surgically correct the hiatal hernia that is linked with the lesion.
